# Proton pump inhibitors reduce survival outcomes in patients treated with capecitabine: meta-analysis

**DOI:** 10.3332/ecancer.2025.1868

**Published:** 2025-03-11

**Authors:** Dina Mohyeldeen, Waleed Arafat

**Affiliations:** Oncology Department, Faculty of Medicine, Alexandria University, Alexandria 21526, Egypt; https://orcid.org/0000-0002-4424-2648

**Keywords:** capecitabine, proton pump inhibitors, omeprazole, hand-foot syndrome, overall survival, progression-free survival, recurrence-free survival

## Abstract

Proton pump inhibitors (PPIs) are widely used over-the-counter drugs. The interaction between capecitabine and PPIs is still ambiguous within the literature, with some discrepancies still being present regarding the risks, or benefits, of their concomitant use. This meta-analysis aims to analyse data from the literature regarding both the risk of PPIs on survival in patients treated with capecitabine, as well as their benefits regarding the incidence of hand-foot syndrome (HFS). A total of 17 studies were included after searching PubMed, Medline and Cochrane until October 2022 for the effect of PPIs on the treatment efficacy and pharmacokinetics, and incidence of HFS. Revman Ver. 5.3 was used for all statistical analyses. Our data showed a significant HFS reduction at a relative risk of 0.77 (95% CI: 0.70–085; *p* < 0.00001) in the PPI-using groups compared to control. Meta-analysis of studies assessing survival, however, showed a reduction in almost all survival aspects, most notably within the recurrence-free survival, with a hazard ratio of 1.75; 95% CI: 1.21–2.53; *p* = 0.003. Individual data incriminating the use of PPIs with capecitabine is quite limited; however, our robust survival data on around 30,000 patients gave significantly worse survival outcomes, particularly in the (neo)adjuvant setting.

## Introduction

Capecitabine is an oral fluoropyrimidine chemotherapeutic drug that is converted to 5-fluorouracil (5FU) through a three-step cascade beginning in the liver and ending within the tumour microenvironment, where the final step takes place, allowing 5FU to exert its anti-tumourigenic effect while sparing the normal tissues [1]. Oral chemotherapy has been emerging as a possible alternative to conventional IV route drugs without diminishing the possible clinical benefit of IV agents [2, 3].

Hand-foot syndrome (HFS), or palmar-plantar erythrodysesthesia, is an adverse event commonly seen with capecitabine, as well as other antineoplastic drugs. It occurs in more than half of the patients taking this treatment and is characterised by distal skin changes ranging from as little as minor oedema and erythema, and up to more severe symptoms such as blistering, desquamation and debilitating pain. In such severe forms, HFS can cause premature treatment discontinuation [4,5].

Although still not completely understood, multiple theories have been implicated in HFS, including the inflammatory pathway; there is no definitive treatment for HFS, and therapy relies mainly on supportive measures. This includes constant hydration, moisturisation and limb elevation. Local creams containing steroids or anti-histaminic formulas can also be used. When it comes to systemic treatments, pyridoxine, steroids and anti-COX-2 drugs are usually used. Most of these treatments exert their effects through their anti-inflammatory properties, with the use of COX-2 inhibitors having prophylactic effects in preventing HFS in up to half of the patients [4–7]

Acid-suppressing drugs are commonly used with capecitabine due to its direct irritating effect on the gastric mucosa, with up to half of oncology patients using one form or another of acid-suppressing therapy [8]. One group of these acid-suppressing drugs is proton pump inhibitors (PPIs). In addition to its widely known anti-acid secretion use in patients with gastritis – which usually occurs with capecitabine [8, 9] – PPIs have complementary anti-inflammatory effects by inhibiting the lysosomal influx of H^+^ ions. This lack of lysosomal acidification can reduce the phagocytic function as well as decrease the adhesion molecule expression and, therefore, the chemotactic abilities of immune cells. PPIs also reduce the production of endothelial and tissue chemotactic cytokines resulting in possible anti-inflammatory effects [10, 11].

A recent study by Hiromoto *et al* [4] has tested this theory in particular as its primary outcome on capecitabine-induced HFS in mice and reported a significant reduction in the severity of the HFS (*p* < 0.05), with it possibly being due to the reduction in the tumour necrosis factor (TNF)-α in the mice limbs (*p* < 0.01). This study also had a retrospective patient-based arm that was also included in the meta-analysis data of HFS on around 60,000 patients [4].

Despite the possible benefits of PPIs on the adverse-event profile of capecitabine, a possible interaction has long been suggested between PPIs and capecitabine, mostly due to the pre-notion that the increase in the gastric PH by the PPIs could possibly affect the gastric fragmentation of the tablet, therefore affecting its rate of absorption [8, 12]. However, multiple pharmacokinetic studies have revoked this theory as assessed in a narrative review assessing this interaction in particular [13].

Multiple studies have retrospectively assessed the effect of PPIs on the efficacy of capecitabine, as analysed in this study. However, the present literature of systematic reviews only assesses the effect of PPIs on treatment efficacy using multiple parameters, which is of course the major factor to consider in this relationship [14, 15]. None of the reviews, however, to our current knowledge, has done a quantitative meta-analysis or concurrently assessed the effect of PPIs on the incidence of HFS or other AE, despite multiple studies reporting such benefits.

In 2019, a systematic review was done by Viñal *et al* [14] specifically to assess the effect of PPIs on the efficacy of capecitabine, with most of the studies in the original review until 2019 being included – save for one study that was excluded due to the nonspecification of the type of acid-suppression therapy used. In addition, when it comes to the studies in the review reported to show significant results, one of the studies included as a conference [16], released the full trial paper in 2020 with additional study subjects [17]. This discrepancy in the results of the studies included in this review, the publishing of newer studies after that, as well as the absence of any meta-analysis studies on this particular interaction between both drugs has created a need for a wholesome review assessing not only the risks but also the benefits of PPIs on the use of capecitabine. This has led to the birth of this systematic review and meta-analysis.

## Methods

In this systematic review and meta-analysis, we aimed to systematically select our included studies by screening some of the major online libraries, including PubMed, Medline, and the Cochrane Libraries, all of which were searched up until October 2022. The search engines were searched for the following keywords ‘((‘proton pump’) OR (omeprazole) OR (lansoprazole) OR (esomeprazole) OR (rabeprazole) OR (pantoprazole)) AND (capecitabine)’

Our meta-analysis adheres to the guidelines provided by the Preferred Reporting Items for Systematic Reviews and Meta-Analyses report (PRISMA guidelines). All studies, whether observational or interventional and of any date, with quantitative data regarding the effect of PPI administration on different survival outcome, capecitabine adverse-event incidence and/or different pharmacokinetic data were all included in the qualitative and quantitative meta-analysis, assuming that the present data are sufficient for at least one aspect of comparison. Study abstracts that lacked a complementary full article were included in case they included sufficient data, while those that had a follow-up published article were dismissed for the sake of the main article. None of the articles were in non-English languages, and therefore, no translations were required. Secondary analysis studies were included, provided that the National Clinical Trial number was checked prior to inclusion to avoid overlap in case of multiple studies analysing the same primary clinical trial.

### Study outcomes

The primary outcome of this review and meta-analysis was the different survival outcomes in the PPI group versus the control group. Different survival outcomes include overall survival (OS) and progression-free survival (PFS) in the metastatic setting, and OS and recurrence-free survival (RFS) or disease-free survival (DFS) in the nonmetastatic setting. The secondary outcomes include the incidence of HFS and diarrhoea, as well as the pharmacokinetic differences between both the PPI and control groups.

### Inclusion criteria

We should mention that during our initial search, we wanted to standardise our inclusion criteria for the definition of ‘PPI use’; however, there was a total lack of standardised criteria for who are ‘PPI-eligible’ patients. We therefore decided to include all studies, including patients concomitantly taking capecitabine and PPIs. More detailed information will be discussed later in the discussion section. We included studies done on human subjects, with participants using any of the following PPIs: omeprazole, lansoprazole, esomeprazole, rabeprazole or pantoprazole. Studies need to have full English text and have available data regarding any of the following capecitabine/PPI interactions: 1) survival outcomes, 2) HFS, 3) diarrhoea or 4) pharmacokinetic interaction.

### Exclusion criteria

Animal studies and studies that lack clear differentiation between PPIs and other acid-suppressing drugs (e.g., H2-receptor antagonists) during analysis were excluded. Studies lacking full English text were also excluded.

### Included studies

In this systematic review, we have included a total of 17 studies out of the original 96 studies brought up from our searching keywords [4, 8, 12, 17–30]. These 17 studies were relevant and had sufficient data and were hence included in the review and meta-analysis. Fourteen of the included studies had data concerning survival – with eight being in the metastatic setting and six in the nonmetastatic setting – nine had information regarding the effect on HFS incidence and three regarding the effect on pharmacokinetics.

**Figure 1.**
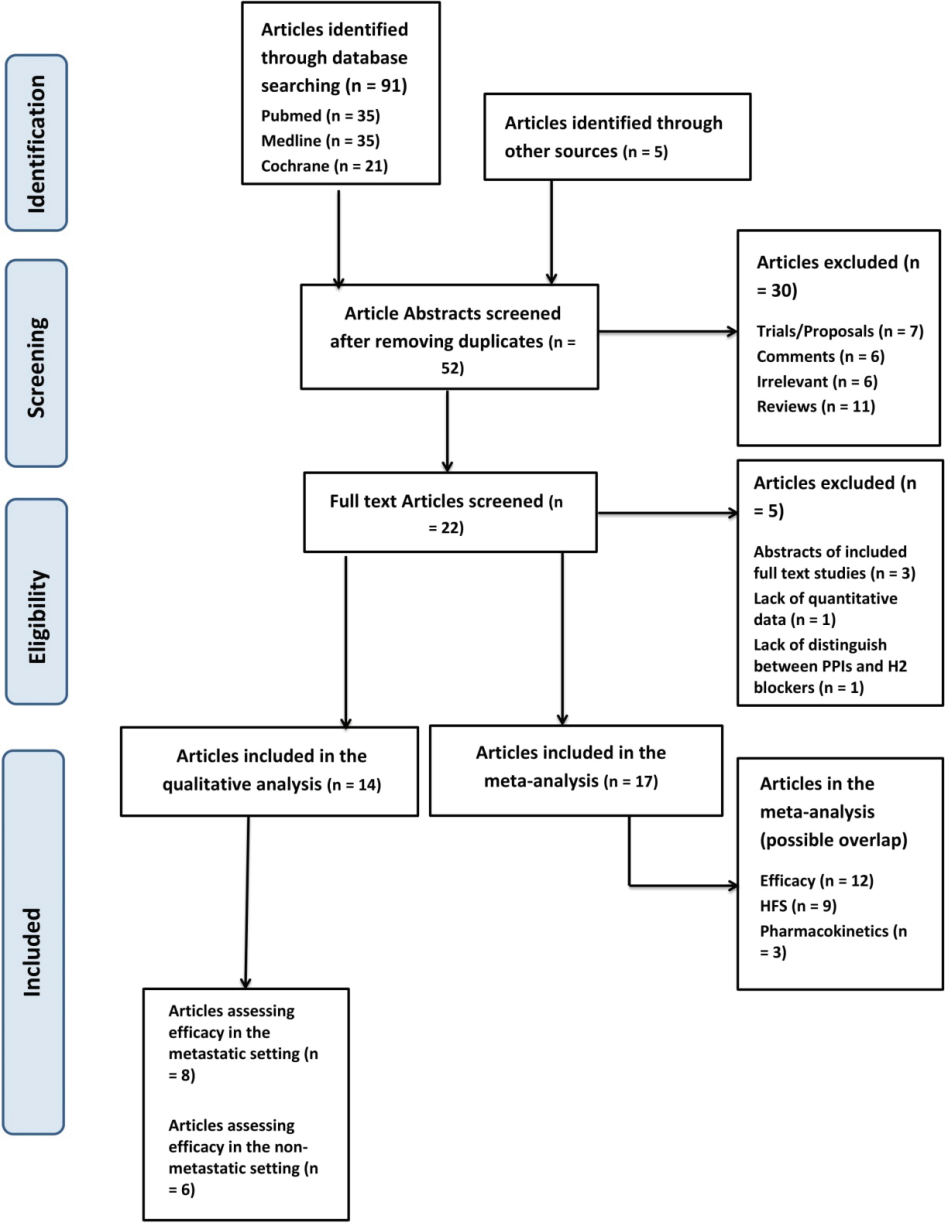
PRISMA flow chart in search for studies assessing the relationship between PPIs and capecitabine. PPIs = proton pump inhibitors; HFS = hand-foot syndrome; PRISMA = preferred reporting items for systematic reviews and meta-analyses.

Regarding the meta-analysis, 12 out of the 14 studies included detailed data regarding the hazard ratios of PPIs on the efficacy of capecitabine and were therefore included in the meta-analysis of the effect of PPI on the OS, RFS, PFS and DFS (Figure 1).

### Statistical analysis

We performed our meta-analysis on the data extracted from the included studies through Revman Ver. 5.3. All of the included forest plots were standardised to favour the PPI arm of the included studies on the left and favour the control group on the right. In some cases where the sample was heterogeneous – *p*-value of heterogeneity >0.05 and *I*^2^ > 50% – then the statistical was changed to ‘Random Model’ to account for this heterogeneity.

Given the presence of some discrepancy in the units used to assess the different pharmacokinetic parameters, conversion measures were needed to standardise the data in order to be able to analyse it correctly. The area under the curve **(**AUC**)** was standardised to μM h/L, with standard deviation (SD) as the measure of data dispersion. Therefore, the ng h/mL unit – in the van Doorn *et al* [30] study – was converted to μM h/L by dividing the AUC (ng h/mL) by the molecular weight of the measured compound [31] (given the molecular weight of capecitabine, 5’ DFUR and 5FU is 359.35, 246.19 and 130.078, respectively [32–35]). The coefficient of variation (CV %) of that AUC was then converted to an SD by multiplying the CV% by the mean and dividing the resulting number by 100.

The Cmax was standardised to μM/L. Therefore, the Cmax in the study by Roberto *et al* [16, 17] was converted from its original unit of μg/mL using the molarity and concentration calculator provided by ‘*Novus Biologicals*’ [36], by multiplying the given Cmax by 1**,**000, setting the volume to litres and adding the molecular weight of the corresponding molecule as previously stated [32–35]. The SD could then be easily calculated.

Both the Tmax and the T1/2 parameters in the study by van Doorn *et al* [30] were supplied as medians and interquartile ranges. Therefore, they were converted to the standard mean and SD through a personal Excel tool made based on the equations provided by Wan *et al* [37].

## Results

### Individual qualitative study assessment of the effect of PPI on PFS/RFS and OS

A total of 14 studies addressed this issue (in breast of gastrointestinal malignancies or both), 8 of which were retrospective studies, three that were secondary analyses and 3 that were clinical trials – either animal or human studies. Out of these studies, 8 assessed patients with metastatic cancer on capecitabine (Supplementary File 1 Table 1 [12, 17, 19–23]), and six assessed nonmetastatic patients in the neo- or adjuvant settings (Supplementary File 1 Table 2 [8, 24–28]).

**Figure 2.**
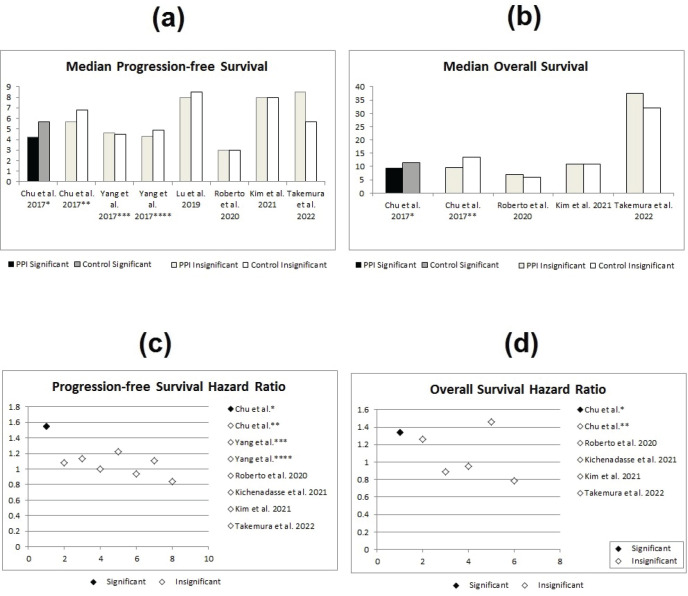
Statistical effect of PPIs on metastatic disease; red studies indicate nonsignificant differences, while blue studies indicate significant differences. PFS = progression-free survival, OS = overall survival, * = CapeOx arm in Chu et al [12]’s study, ** = CapeOx and lapatinib arm in Chu et al [12]’s study, *** = gastrointestinal cancer arm in Yang et al [19]’s study and **** = breast cancer arm in Yang et al [19]’s study. (a) Median PFS in PPI versus control group. (b) Median OS in PPI versus control group. (c) Median PFS hazard ratios for PPI versus control group. (d) Median OS hazard ratios for PPI versus control group.

Out of the 14 studies comparing treatment efficacy with the concomitant use of PPIs versus control, only three studies showed significant differences between both groups [8, 12, 25]. These three studies were either in the retrospective analyses [8, 25] or the secondary analysis category [12] – none were clinical trials. The first one of these three was the study done by Chu *et al* [12], where there appeared to be significant difference in the hazard ratios for both PFS (HR = 1.55; 95% CI: 1.29–1.81; *p* < 0.001) and OS (HR = 1.34; 95% CI: 1.06–1.62; *p* = 0.04) in the CapeOx only arms, with the lapatinib arms showing no significant difference (Figure 2).

**Figure 3.**
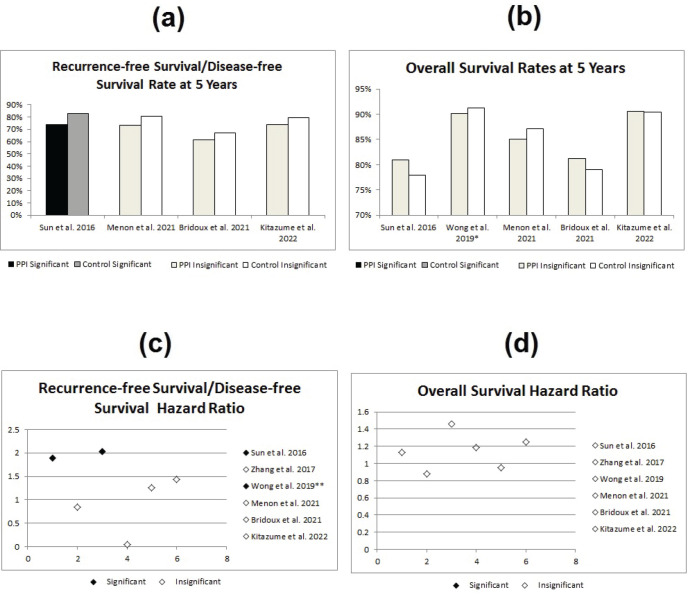
Statistical effect of PPIs on metastatic disease; red studies indicate nonsignificant differences, while blue studies indicate significant differences. (a) DFS/RFS rates at 5 years in PPI versus control group. (b) OS rates at 5 years in PPI versus control group. (c) RFS/DFS hazard ratios for PPI versus control group. (d) OS hazard ratios for PPI versus control group. RFS = recurrence-free survival, DFS = disease-free survival, OS = overall survival, * = Wong et al [25]’s OS rates are at 3 years and ** = Wong et al [25]’s significant results were lost when the study accounted for different confounders.

The second study, showing significant differences between both groups, was the study done by Sun *et al* [8] which showed statistically significant lower RFS in the PPI group (HR = 1.89; 95% CI: 1.07–3.35; *p* = 0.03). In addition, the study by Wong *et al* [25] has also shown a statistically significant double the RFS in the control group (HR 2.03; 95% CI 1.06–3.88; *p* = 0.033, see Figure 3).

It is also worth mentioning that in Wang *et al* [18]’s study, which is later included in the meta-analysis of the effect of PPIs on the incidence of HFS, the authors reported additional information regarding the possible interaction between PPIs and capecitabine in the CapeOx arm and reported no significant differences in either the PFS (*p* = 0.52) or the OS (*p* = 0.98) – with no detailed data regarding the exact hazard ratios included. This study was not included in the qualitative or meta-analysis figures and was only included in the supplementary File 1 Table 1 provided.

### Meta-analysis of the effect of PPIs on the efficacy of capecitabine

**Figure 4.**
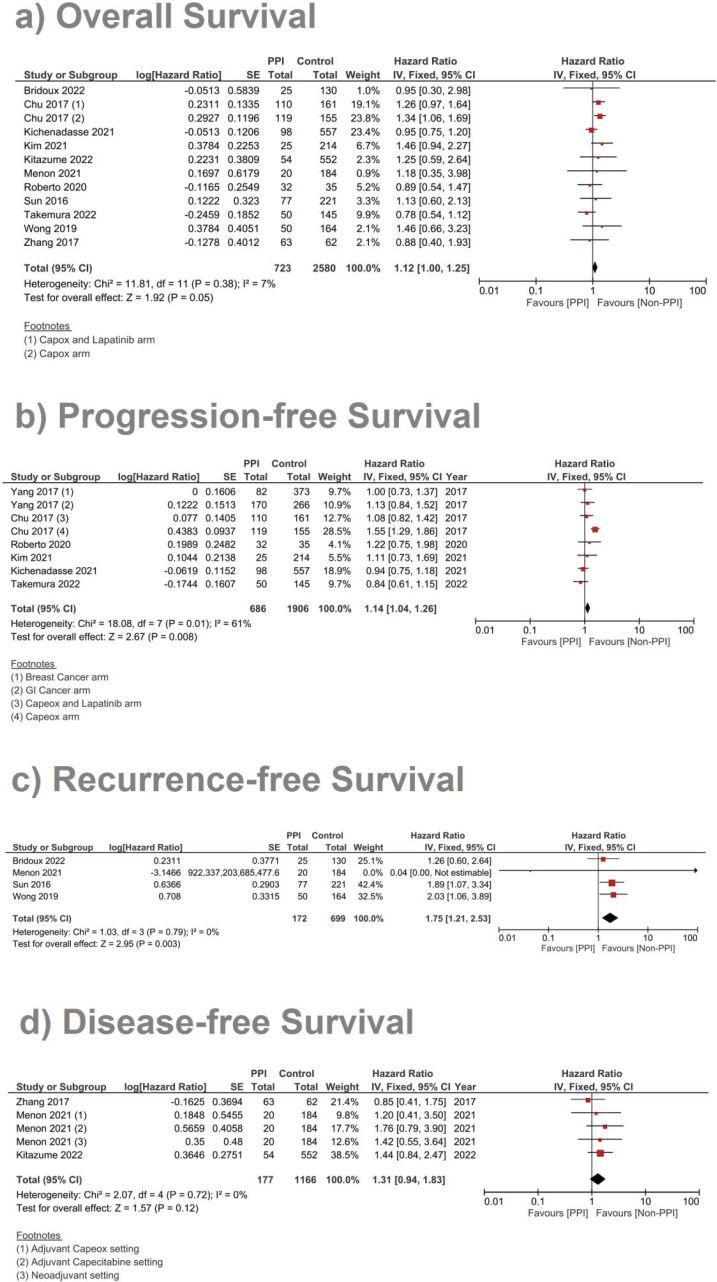
Forest plot of the effect of PPIs on three survival outcomes as predicted by the risk ratio (significance at *p*-value <0.05). a) Assessment of the OS. b) Assessment of the PFS. c) Assessment of the RFS.

**Supplementary Figure 1.**
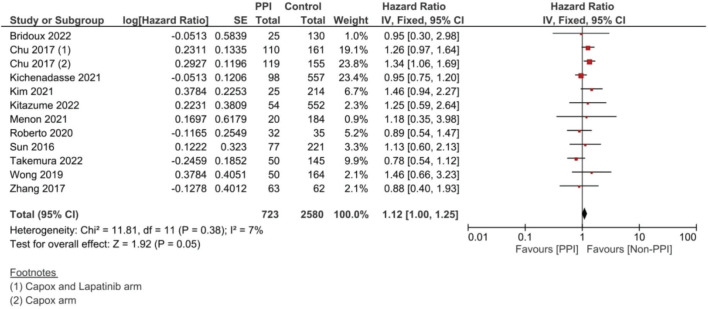
Unadjusted OS hazard ratio pooled analysis.

**Supplementary Figure 2.**
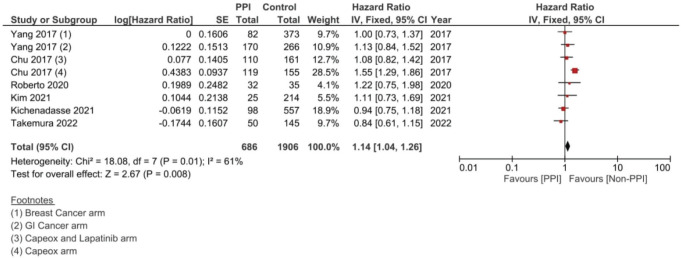
Unadjusted PFS hazard ratio pooled analysis.

**Supplementary Figure 3.**
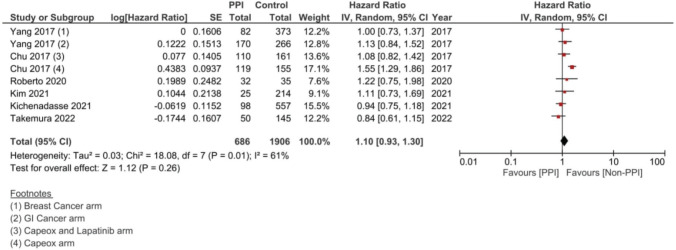
Unadjusted PFS hazard ratio pooled analysis using ‘Random Model’ analysis.

**Supplementary Figure 4.**
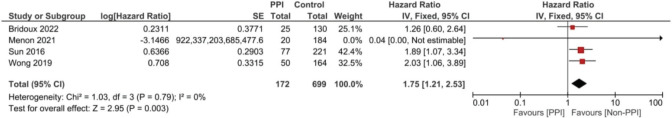
Unadjusted RFS hazard ratio pooled analysis.

**Supplementary Figure 5.**
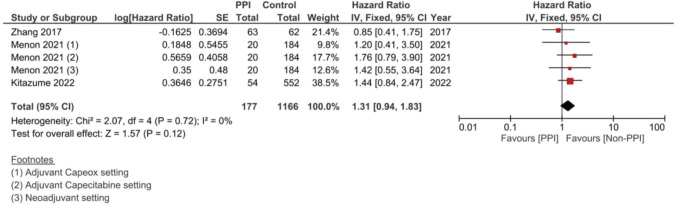
Unadjusted DFS hazard ratio pooled analysis.

**Supplementary Figure 6.**
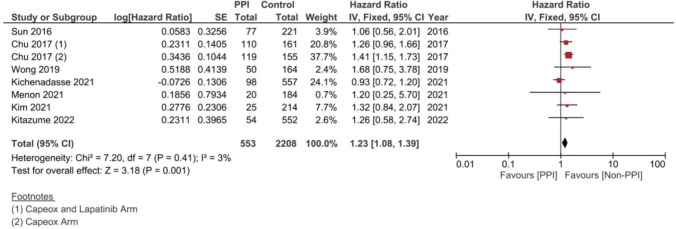
Adjusted OS hazard ratio pooled analysis.

**Supplementary Figure 7.**
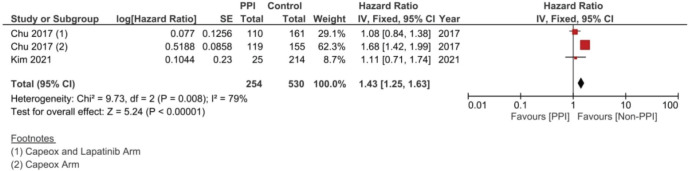
Adjusted PFS hazard ratio pooled analysis.

**Supplementary Figure 8.**
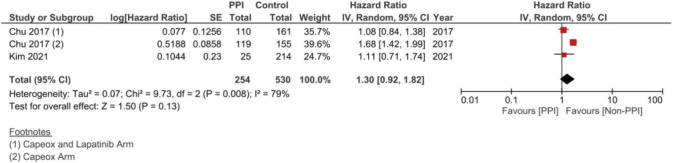
Adjusted PFS hazard ratio pooled analysis using ‘Random Model’ analysis.

**Supplementary Figure 9.**
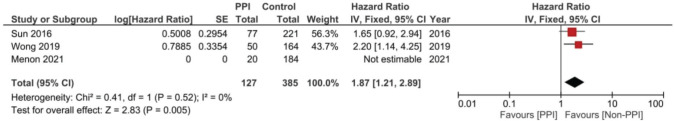
Adjusted RFS hazard ratio pooled analysis.

**Supplementary Figure 10.**
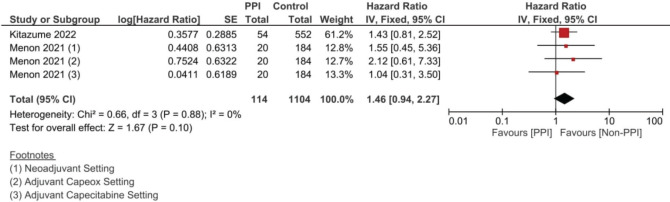
Adjusted DFS hazard ratio pooled analysis.

A complementary meta-analysis for the OS, PFS and/or RFS was done individually for studies, including hazard ratios of such outcomes, using inverse variance (Supplementary File 2 Figures 1–10). With regard to OS, 11 studies were included using the unadjusted HRs (Figure 4). The analysis found a statistically significant effect on the OS, with a pooled HR of 1.12, 95% CI 1.00–1.25 and *p* = 0.05 (Figure 4a). On using the adjusted HR for analysis, the pooled HR became 1.23, with a 95% CI of 1.08 and 1.39, and *p* = 0.001.

In the case of studies assessing metastatic malignancies, the pooled HR of the PFS from six studies showed a statistically significant difference at 1.14, 95% CI 1.04–1.26 and *p* = 0.008 (Figure 4b). However, on accounting for the present sample heterogeneity – *I*^2^ 61% and *p*-value of heterogeneity 0.03 – through using a random model analysis, this significance was lost; HR = 1.10, 95% CI 0.93–1.30 and *p* = 0.26; Supplementary File 2 Figure 3. The adjusted pooled HR for PFS was also significant at 1.43, 95% CI 1.25 and 1.63 and *p* < 0.00001. Yet, on doing random model analysis to account for sample heterogeneity, *I*^2^ 79%, the significance of the effect on the adjusted PFS was once again lost (HR = 1.30, 95% CI 0.92–1.82 and *p* = 0.13; Supplementary File 2 Figure 8).

Meanwhile, in studies assessing patients in the (neo)adjuvant setting, six studies were assessed, and the four studies assessing RFS showed the strongest association at a pooled HR of 1.75, 95% CI: 1.21–2.53 and *p* = 0.003; Figure 4c, and a pooled adjusted HR of 1.87, 95% CI 1.21–2.89 and *p* = 0.005. A meta-analysis was also done on DFS; yet, no significant difference was found in the unadjusted (HR: 1.31; 95% CI: 0.94–1.83; *p* = 0.12, Figure 4d) or confounder-adjusted setting (HR: 1.46; 95% CI: 0.94–2.27; *p* = 0.10).

### Meta-analysis of the effect of PPIs on the incidence of HFS

**Figure 5.**
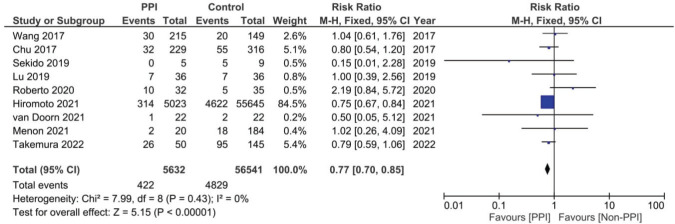
Forest plot of the effect of PPIs on the incidence of capecitabine-induced HFS as predicted by the risk ratio (significance at *p*-value <0.05).

**Supplementary Figure 11.**
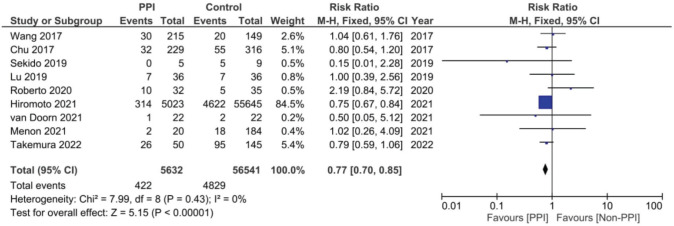
Risk ratio of HFS pooled analysis.

**Supplementary Figure 12.**
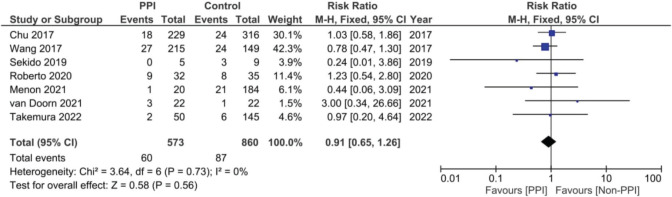
Risk ratio of diarrhoea pooled analysis.

A total of nine studies, three of which were clinical trials, were included in the meta-analysis assessing the relationship between the concomitant administration of PPI with capecitabine and the incidence of HFS [4, 12, 17, 18, 20, 26, 29, 30]. The analysis showed statistically significant lower relative risk by around 23% in the PPI group when compared to the control group (RR: 0.77; 95% CI: 0.70 and 0.85; *p* < 0.00001, Figure 5, Supplementary Figure 11.). There was no statistical significance; however, regarding the use of PPI and the incidence of diarrhoea after analysing six of the included studies (RR: 0.95; 95% CI: 0.65 and 1.26; *p* 0.56, Supplementary File 2 Figure 12 [12, 17, 18, 26, 29, 30]).

The study done by Takemura *et al* [23] has also reported additional data regarding HFS, where they found a greater difference between both groups regarding the incidence of HFS when the HFS grade was adjusted to ≥Grade 2, with significantly lower HFS events reported in the PPI group (18% versus 43% in the PPI versus non-PPI groups, *p* = 0.001 [23]). In addition, the PPI group reported a lack of pre-mature capecitabine termination due to HFS compared to the non-PPI group (14%), as well as longer time of onset to HFS (reaching up to 20 months in the PPI group with a median of 1.4 months, compared to up to 9 months in the non-PPI-using group with a median of 2.2 months).

### Pharmacokinetics

**Supplementary Figure 13.**
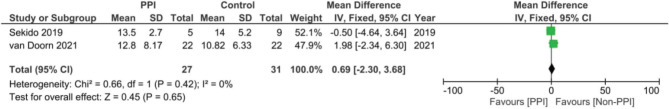
AUC of capecitabine pooled analysis (μM h/L).

**Supplementary Figure 14.**
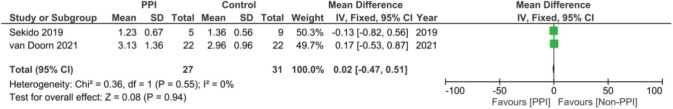
AUC of 5FU pooled analysis (μM h/L).

**Supplementary Figure 15.**
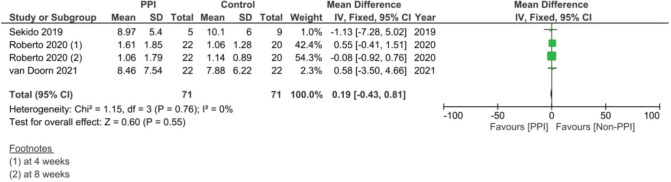
Maximal concentration of capecitabine pooled analysis (μM/L).

**Supplementary Figure 16.**
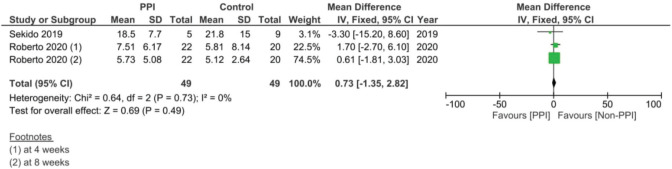
Maximal concentration of 5′-deoxy-5-fluorouridine (5DFUR) pooled analysis (μM/L).

**Supplementary Figure 17.**
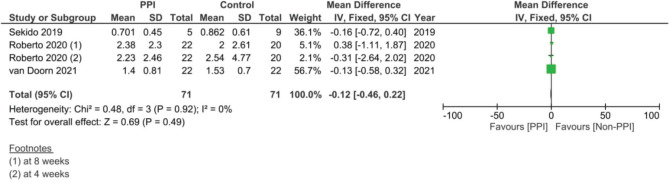
Maximal concentration of 5FU pooled analysis (μM/L)

**Supplementary Figure 18.**
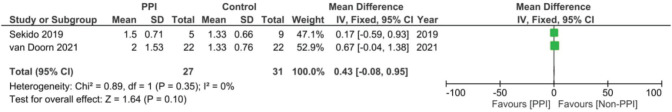
Time to reach maximal concentration of capecitabine pooled analysis (hours).

**Supplementary Figure 19.**
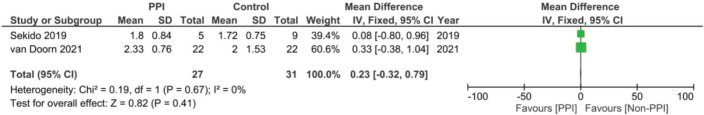
Time to reach maximal concentration of 5FU pooled analysis (hours).

**Supplementary Figure 20.**
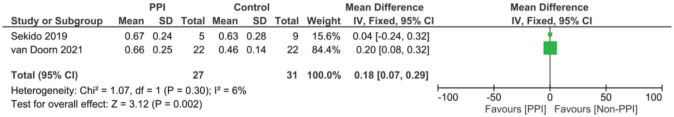
Half-life time of capecitabine (hours).

In assessing the effect of PPIs on the pharmacokinetics – including the AUC, Cmax, Tmax and T1/2 – of capecitabine, three studies were included [17, 29, 30]. However, none of the assessed parameters showed statistically significant results on doing individual meta-analysis for each parameter (Supplementary File 2 Figures 13–20).

## Discussion

Capecitabine is an oral 5FU pro-drug fluoropyrimidine chemotherapeutic agent [1]. Oral chemotherapy has been emerging as a more convenient alternative for conventional IV route drugs without diminishing the possible clinical benefit achieved by the IV agents [2, 38, 39]. In a questionnaire done on around 400 patients who have previously received both oral and IV chemotherapy regimens, a major preference for the oral route was seen in around three-fourths of the patients. This preference is mostly due to the lower alteration of daily life routine, less hospital waiting time, less IV-related complications and less worry about IV access-related difficulties [38].

One of the most commonly associated adverse events associated with capecitabine is gastrointestinal upset, commonly treated with PPIs or other forms of acid-suppressing drugs [9]. However, PPIs have long been avoided with capecitabine due to some evidence of interference with its pharmacokinetics and efficacy [6, 7]. Some evidence suggests that PPIs interfere with the action of capecitabine by raising the gastric PH and hence interfering with the absorption – and therefore efficacy – of capecitabine. Although a review recently done by Cheng *et al* [13] has reported the lack of evidence regarding this notion, given that capecitabine tablets were proven to be able to dissolve over multiple PH degrees**,** physicians are still precautious regarding concomitant use to this day**.** The suggested interaction between PPI and capecitabine is mostly due to the pre-notion that the increase in the gastric PH by the PPIs could possibly affect the gastric fragmentation of the tablet and therefore affect its rate of absorption [8, 12]. However, capecitabine tablets can dissolve over multiple PH degrees ranging from the highly acidic spectrum up to an almost neutral environment [13], and therefore, the average gastric pH while on PPI – around 4 – is not sufficient to significantly affect the ionization, and absorption, of capecitabine.

PPIs have long been studied for possible extra-acid-suppression benefits. They have proven to bear anti-inflammatory as well as possible anti-resistance benefits in the case of multidrug-resistant cancers [10, 40]. However, one of the recently studied benefits includes a recent study by Hiromoto *et al* [4] that has reported a significant reduction in the severity of the HFS (*p* < 0.05), possibly due to the reduction in the TNF-α in the mice limbs (*p* < 0.01). This study also had a retrospective patient-based arm that was also included in the meta-analysis data of HFS and has reported a significant reduction in the HFS in people who were taking concomitant PPI – with an odds ratio of 0.74 in favour of PPI use [4].

Given the contradicting results regarding the benefits and the risks of using PPIs concomitantly with capecitabine, we have tried to meticulously assess both in our meta-analysis, to account for the already-present discrepancy within the literature.

Qualitative assessment of each included study regarding different safety outcomes revealed significant findings in 3 out of the 14 studies. However, most of these studies, 8 of the 14, are retrospective in origin, with another three being secondary analyses of prior trials, making their scientific evidence of lower value when compared to actual primary clinical trials with confounder control [41].

Another possible drawback of the included retrospective studies is that all of the study data were based only on drug dispensal data, with some studies including patients in the PPI group if they received PPIs at any point during treatment [8, 25], therefore exposing these studies to a form of selection bias. Another discrepancy is seen in the study by Chu *et al* [12] which showed significant differences only in the incidence within the CapeOx-only arm, while the CapeOx/lapatinib arm showed no difference with the use of PPIs. This might raise questions regarding the validity of such results, given that lapatinib does not cause HFS in the first place [42]. In addition, in the study by Wong *et al* [25], confounder adjustment reversed the statistical significance of the effect of PPIs on the RFS (HR: 2.20; 95% CI: 1.14–4.25; *p* = 0.18). This is not to in any way suspect the validity or credibility of the studies’ methodologies or significant findings, but to point out the common possible limitations, like all of the data obtained from retrospective studies [41, 43].

To this end, the data from our meta-analysis concerning the efficacy has shown that the concomitant use of PPIs was associated with a decline in the OS (HR 1.12 and *p* = 0.05), PFS (HR 1.14 and *p* = 0.008) and RFS (HR 1.75 and *p* = 0.003). Yet, the significance in the PFS effect, both the unadjusted and adjusted hazard ratios, was abolished by using the random effect analysis to account for sample heterogeneity – *I*^2^ at 61% and 79%, respectively. The RFS was the parameter scoring the highest HR in response to concomitant PPI administration (HR = 1.75 and *p* = 0.003), which was even higher when adjusted for confounding factors; to reach 1.89 and *p* = 0.005, Supplementary File 2 Figure 9. DFS reported no significant differences between both groups: Figure 4d and Supplementary File 2 Figures 5 and 10.

When it comes to safety prognosis, PPIs were associated with a lower incidence of HFS, RR 0.77 and *p* < 0.00001 (Figure 5). These findings were in line with the findings of the study by Hiromoto *et al* [4] which contributed to the majority of the weight of this analysis at a sample size as large as 60,668 patients with an RR of 0.75.

When it comes to the effect of PPIs on the incidence of capecitabine-induced diarrhoea, there was also no reduction in the rates of diarrhoea with PPI use. We hypothesise that first, this lack of improvement may be attributed to the fact that chronic PPI use has already been suggested to cause diarrhoea – independent from capecitabine use [44, 45]. This has been suggested to occur either due to chronic acid suppression with clostridium difficile overgrowth or due to direct PPI-associated mucositis (colitis) [44] – which is already one major mechanism of capecitabine-associated diarrhoea [46]. Second, we suggest that the lack of possible exacerbation of capecitabine-induced diarrhoea with the use of PPI can mostly be attributed to the limited number of studies assessing this particular interaction – with a total sample size of less than 1,500 patients for the total of seven included studies, as illustrated in Supplementary File 2 Figure 12, which is much lower when compared to other addressed aspects like HFS or survival analysis. In conclusion, not knowing how long did include patients used PPIs, and if they used them on a chronic basis or not (to cause PPI-induced diarrhoea), coupled with the smaller sample size, suggests that we still cannot say that PPIs have no impact on the incidence of capecitabine-induced diarrhoea – whether in a positive or a negative way.

Finally, when we analysed the pharmacokinetic overlap between both drugs, no significant correlation was found. The lack of significant difference could in our opinion be attributed to the lower number of studies, as well as the different units and times each study measured the PK parameters after the start of therapy, causing high variations in the levels between the studies (Supplementary File 2 Figures 13–20). On the one hand, the study by Sekido *et al* [29] measured the plasma levels on the first day of the first cycle, while the study by Roberto *et al* [17] measured them at weeks 4 and 8 and the study by van Doorn *et al* [30] measured them on day 8 of each phase [30]. This could have actually created major discrepancies in the pharmacokinetic comparison across studies.

Despite the possible detrimental effects of PPIs on survival, our findings concerning the incidence of HFS are in our opinion quite promising, even if not directly. The results of our analysis might open the doors for future studies to fully discover and make use of the exact mechanism by which PPIs reduce HFS. Therefore, could this open the doors for the use of anti-TNF agents in patients taking capecitabine? particularly given the fact that multiple studies have reported the lack of cancer development or progression in patients diagnosed with IBD – with even potential benefits in osseous metastases as well as overcoming treatment resistance to multiple agents [47–50].

Possible limitations in our meta-analysis include the retrospective nature of most studies included for survival analysis, which might have contributed to either diminished or exaggerated results. In addition, different follow-up durations in the studies, and notable discrepancies in pharmacokinetic data, including the time of assessment since beginning treatment as well as the different measurement units, are also additional limitations that might contribute to analysis errors while assessing for pharmacokinetic interaction. This discrepancy and lack of standardization are mostly attributed to the retrospective nature of most of our included studies. Most of these studies assess the drug interaction between capecitabine and PPIs through healthcare records [12, 23, 25, 27, 28], possibly to avoid clinical trials with possible harm to patients on capecitabine. Obtaining records retrospectively does not really give insight into patients’ true PPI intake frequency or dosage compliance. However, we should mention that multiple included retrospective studies considered a patient eligible for inclusion in the ‘PPI’ category if they concomitantly received PPIs for a minimum concomitant duration of 20% [8, 22], but still, many studies failed to put clear criteria for what exactly they considered a ‘PPI-eligible patient’ [18, 19] or even considered a single, or any, prescription of PPI as being eligible to enter the PPI group [26, 27]. Given this lack of standardised patient selection criteria, we recommend waiting for further interventional trials to assess this relationship using standardised PPI dosing and assessment criteria before putting assuming any interaction – or lack thereof.

Individual data incriminating the use of PPIs with capecitabine are quite limited – with possible confounders and validity threats in multiple studies seen during our qualitative assessment, due to study design issues, as previously mentioned. However, given the present fear of conducting a clinical trial in case of a positive association, this only leaves us with the possibility of doing a meta-analysis in order to get a better insight into these contradictory findings, as done in this paper.

## Conclusion

In conclusion, our meta-analysis on this large population – reaching as many as 3**,**303 patients in the survival analyses and reaching 62,173 patients in the HFS incidence assessment – has reported both beneficial and detrimental interactions with capecitabine. PPIs are associated with a lower incidence of HFS compared to the control group, with a 33% relative risk reduction in the incidence of HFS. However, regarding possible survival risk, PPIs have shown statistically significant worse treatment outcomes in all aspects, save for the DFS, with a much greater impact on the RFS in nonmetastatic cases with up to 75% higher relative risk of recurrence at an HR of 1.75, *p* = 0.003 and increasing up to 87% increased risk when adjusted for confounders. This should in turn warrant caution and awareness on the possible risks of concurrent use of PPIs along with capecitabine, with extra-caution and meticulous history taking in patients taking capecitabine in the (neo)adjuvant setting due to the much higher impact on patient survival.

## List of abbreviations

5FU, 5-fluorouracil; AUC, Area under the curve; CV, Coefficient of variation; DFS, Disease-free survival; HFS, Hand-foot syndrome; NCT, National Clinical Trial, OS: Overall survival; PFS, Progression-free survival; PPIs, Proton pump inhibitors; PRISMA, Preferred Reporting Items for Systematic Reviews and Meta-Analyses; RFS, Recurrence-free survival; SD, Standard deviation.

## Conflicts of interest

We report no conflicts of interest for this meta-analysis.

## Funding

This research did not receive any specific grant from funding agencies in the public, commercial or not-for-profit sectors.

## Full supplementary data of the review and meta-analysis results

**(A) Efficacy meta-analysis**

**Supplementary Table 1.** Median-time and hazard ratios for PFS and OS differences between PPI and non-PPI users among METASTATIC cancer patients treated with capecitabine; significance at *p* < 0.05.

Study	Study type	*N* PPI	Exposed arm	*N*	Control arm	Median PFS PPI versus Ctrl	HR	95% CI	*p*-value	Median OS PPI versus Ctrl	HR OS	95% CI	*p*-value	Verdict
**Chu *et al* [12]**	Secondary analysis	119	CapeOx + PPI	155	CapeOx	4.2 versus 5.7 Months	1.55	1.29–1.81	<0.001	9.2 versus 11.3 Months	1.34	1.06–1.62	0.04	Significant for PFS and OS
		110	CapeOx + Lapatinib + PPI	161	CapeOx + Lapatinib	5.7 versus 6.8 Months	1.08	0.82–1.34	0.54	9.6 versus 13.6 Months	1.26	0.97–1.55	0.1	**Insignificant** for PFS and OS
**Wang *et al* [18]**	Primary trial (*In vitro* + Animal)	215	CapeOx + PPI	149	CapeOx	NA	NA	NA	0.52	NA	NA	NA	0.98	Insignificant for PFS and OS
**Yang *et al* [19]**	Primary trial	170	Any capecitabine regimen + Ome./Panto. (Gastrointestinal cancer)	266	Any Capecitabine regimen (Gastrointestinal cancer)	140 versus 136 Days	1.13	0.84–1.50	Insignificant	NA	NA	NA	NA	Insignificant for PFS
		82	Any capecitabine regimen + Ome./Panto. (Breast cancer)	373	Any capecitabine regimen (Breast cancer)	132 versus 150 Days	1	0.73–1.36	Insignificant	NA	NA	NA	NA	Insignificant for PFS
**Lu *et al* [20]**	Retrospective study	36	Any capecitabine regimen + Ome.	36	Any capecitabine regimen	8 versus 8.5 Months	NA	NA	*p* >0.05	NA	NA	NA	NA	Insignificant for PFS
**Roberto *et al* [17]**	Primary trial	32	Metronomic Capecitabine + Rabe	35	Metronomic capecitabine	3 versus 3 Months	1.22	0.75–2.00	0.42	7 versus 6 Months	0.89	0.54–1.48	0.664	Insignificant for PFS and OS
**Kichenadasse *et al* [21]**	Secondary analysis	98	CapeOx + PPI	557	CapeOx	NA	0.94	0.75–1.17	Insignificant	NA	0.95	0.75–1.22	Insignificant	Insignificant for PFS and OS
**Kim *et al* [22]**	Secondary analysis	25	Xeliri +PPI	214	Xeliri	8 versus 8 Months	1.11	0.73–1.70	0.62	16 versus 16 Months	1.46	0.94–2.26	0.093	Insignificant for PFS and OS
**Takemura *et al* [23]**	Retrospective study	50	Capecitabine + PPI +/- targeted therapy	145	Capecitabine +/- targeted therapy	8.5 versus 5.7 Months	0.84	0.613–1.149	0.287	37.5 versus 32.1 Months	0.782	0.544–1.126	0.210	Insignificant for PFS and OS

N: Sample size; PPI: Proton pump inhibitors; Ctrl: Control group; PFS: Progression-free survival; OS: Overall survival; RFS: Recurrence-free survival; DFS: Disease-free survival; HR: Hazard ratio; CI: Confidence interval; **CapeOx**: Capecitabine + Oxaliplatin; Ome: Omeprazole; Panto: Pantoprazole; Rabe: Rabeprazole

**Supplementary Table 2.** Median-time and hazard ratios for DFS/RFS and OS differences between PPI and non-PPI users among NON-METASTATIC cancer patients treated with capecitabine; significance at *p* < 0.05.

Study	Study type	*N* PPI	Exposed arm	*N*	Control arm	DFS/RFS rates PPI versus Ctrl	*p*-value	OS rate PPI versus Ctrl	*p*-value	HR RFS/DFS	95% CI	*p*-value	HR OS	95% CI	*p*-value	Verdict
**Sun *et al* [8] (Adjuvant)**	Retrospective study	77	Capecitabine monotherapy + PPI	221	Capecitabine monotherapy	**RFS at 5 years:** 74% versus 83%	0.03	**OS at 5 years:** 81% versus 78%	0.7	**RFS:** 1.89	1.07–3.35	0.03	1.13	0.60–2.14	0.7	**Significant** for RFS ONLY
**Zhang *et al* [24] (Neoadjuvant)**	Retrospective study	63	CapeOx + Ome	62	CapeOx	**NA**	NA	**NA**	NA	**DFS:** 0.85	0.43–1.69	0.66	0.88	0.42–1.85	0.75	**Insignificant** for DFS and OS
**Wong *et al* [25] (Adjuvant)**	Retrospective study	50	CapeOx + PPI	164	CapeOx	NA	NA	**OS at 3 years:** 90.1% versus 91.2%	0.345	**RFS:** 2.03	1.06–3.88	0.033	1.46	0.66–3.24	0.348	**Significant** for RFS ONLY** (Lost after factor adjustment)**
**Menon *et al* [26] (Neoadjuvant then adjuvant)**	Primary trial	20	Any capecitabine regimen + PPI	184	Any capecitabine regimen	**DFS at 36 M:** 84.4% versus 85% **DFS at 60 M:** 73.2% versus 80.90%	0.463	**OS at 36 M:** 85% versus 90.6% **OS at 60 M:** 85% versus 87.1%	0.84	**RFS:** 0.043	0.00–6.9E+3	0.606	1.185	0.353–3.974	0.783	**Insignificant** for RFS, DFS and OS
**Bridoux *et al* [27] (Neoadjuvant)**	Retrospective study	25	Capecitabine or CapeOx + PPI	130	Capecitabine or CapeOx	**RFS at 36M:** 61.5% versus 71.6% **RFS at 60M** 61.5% versus 66.9%	0.54	**OS at 36M:** 87.4% versus 90.6% **OS at 60M:** 81.2% versus 79%	0.93	**RFS:** 1.26	0.61–2.6	0.54	0.95	0.33–2.76	0.93	**Insignificant** for RFS and OS
**Kitazume *et al* [28] (Adjuant)**	Retrospective study	54	Capecitabine or CapeOx + PPI	552	Capecitabine or CapeOx	**DFS at 60M:** 73.8% versus 79.6s%	NA	**OS at 60M:** 90.5% versus 90.4%	NA	**DFS:** 1.44	0.84–2.47	0.185	1.25	0.6–2.6	0.558	**Insignificant** for DFS and OS

N: Sample size; PPI: Proton pump inhibitors; Ctrl: Control group; PFS: Progression-free survival; OS: Overall survival; RFS: Recurrence-free survival; DFS: Disease-free survival; HR: Hazard ratio; CI: Confidence interval; **CapeOx**: Capecitabine + Oxaliplatin; Ome: Omeprazole; Panto: Pantoprazole; Rabe: Rabeprazole

### Table of contents: supplementary figures

1. Efficacy meta-analysis 16
  1. Unadjusted hazard ratio analysis 19
    - Unadjusted OS hazard ratio 19
    - Unadjusted PFS hazard ratio 19
    - Unadjusted PFS hazard ratio using “Random Model” analysis 20
    - Unadjusted RFS hazard ratio 20
    - Unadjusted DFS hazard ratio 20
  2. Adjusted hazard ratio analysis 21
    - Adjusted OS hazard ratio 21
    - Adjusted PFS hazard ratio 21
    - Adjusted PFS hazard ratio using “Random Model” analysis 21
    - Adjusted RFS hazard ratio 22
    - Adjusted DFS hazard ratio 22
2. Adverse event meta-analysis 22
  1. HFS 22
    - Risk ratio of HFS pooled analysis 22
  2. Diarrhea 23
3. Pharmacokinetics meta-analysis 23
  1. Area under the concentration-time curve (AUC) 23
    - AUC of Capecitabine 23
    - AUC of 5FU 23
  2. Maximal concentration (Cmax) 24
    - Maximal concentration of capecitabine 24
    - Maximal concentration of 5´-deoxy-5-fluorouridine (5DFUR) 24
    - Maximal concentration of 5FU 24
  3. Time to reach maximal concentration (Tmax) 25
    - Time to reach maximal concentration of capecitabine 25
    - Time to reach maximal concentration of 5FU 25
  4. Half-life (T1/2) 25
    - Half-life time of capecitabine 25

1. Unadjusted hazard ratio analysis (measured using hazard ratio; with a null value of 1) Statistical significance at *p*-value < 0.05

2. Adjusted hazard ratio analysis (measured using hazard ratio; with a null value of 1) Statistical significance at *p*-value < 0.05

**(B) Adverse-event meta-analysis**

1. HFS; measured using risk ratio; with a null value of 1 Statistical significance at *p*-value <0.05

2. Diarrhoea Statistical significance at *p*-value < 0.05

**(C) Pharmacokinetics meta-analysis**

1. Area under the concentration-time curve (AUC; measured in μmol h/L) Statistical significance at *p*-value < 0.05

2. Maximal concentration (Cmax; measured in μmol/L) Statistical significance at *p*-value < 0.05

3. Time to reach maximal concentration (Tmax; measured in hours) Statistical significance at *p*-value <0.05

4. Half-life (T1/2; measured in hours) Statistical significance at *p*-value < 0.05
